# Evaluation of Upper Airway Width and Facial Height Cephalometric Parameters in Adult Caucasians with Skeletal Class I and Class III Malocclusion

**DOI:** 10.3390/medicina61030463

**Published:** 2025-03-06

**Authors:** George Popa, Dana-Cristina Bratu, Sorin Gheorghe Mihali, Silvia Izabella Pop, Bianca Dragoș, Remus-Christian Bratu, Anca Tudor, Anca Jivănescu

**Affiliations:** 1 Department of Orthodontics II, Orthodontic Research Centre, Faculty of Dental Medicine, “Victor Babes” University of Medicine and Pharmacy of Timisoara, 2 Eftimie Murgu Square, 300041 Timisoara, Romania; popa.george@umft.ro; 2 Department of Prosthodontics, Faculty of Dentistry, “Vasile Goldis” Western University of Arad, 94 Revolutiei Blvd., 310025 Arad, Romania; 3 Department of Orthodontics, Faculty of Dental Medicine, George Emil Palade University of Medicine, Pharmacy, Science, and Technology of Targu Mures, 38 Gheorghe Marinescu Street, 540142 Targu Mures, Romania; silvia.pop@umfst.ro; 4 Research Centre in Dental Medicine Using Conventional and Alternative Technologies, Faculty of Dental Medicine, “Victor Babes” University of Medicine and Pharmacy of Timisoara, 9 Revolutiei 1989 Blvd., 300070 Timisoara, Romania; bianca.roman@umft.ro; 5 Faculty of Dental Medicine, “Victor Babes” University of Medicine and Pharmacy of Timisoara, 2 Eftimie Murgu Square, 300041 Timisoara, Romania; remus.bratu@student.umft.ro; 6 Department of Functional Sciences—Medical Informatics and Biostatistics, Research Centre in Dental Medicine Using Conventional and Alternative Technologies, Faculty of Medicine, “Victor Babes” University of Medicine and Pharmacy of Timisoara, 2 Eftimie Murgu Square, 300041 Timisoara, Romania; atudor@umft.ro; 7 Department of Prosthodontics, Digital and Advanced Technique for Endodontic, Restorative and Prosthetic Treatment (TADERP) Research Centre, Faculty of Dental Medicine, “Victor Babes” University of Medicine and Pharmacy of Timisoara, 2 Eftimie Murgu Square, 300041 Timisoara, Romania; jivanescu.anca@umft.ro

**Keywords:** facial height, upper airway width, cephalometric analysis, skeletal Class I, skeletal Class III

## Abstract

*Background and Objectives*: The main objectives of our study were to assess sexual dimorphism and to compare the facial height, as well as the anteroposterior width of the upper airway, within adult Caucasians diagnosed with skeletal Class I and skeletal Class III malocclusion, based on a number of angular and linear cephalometric parameters. *Materials and Methods*: One hundred lateral cephalograms were selected from orthodontic adult Caucasian patients from western Romania. Several angular parameters (SNA, SNB, ANB, FMA, Y–FH, Ba–S–PNS and NL–ML angles) and linear parameters (total, upper and lower anterior facial height—TAFH, UAFH, LAFH; total posterior facial height—TPFH) were analysed for each case. The upper airway width parameters included the width of the nasopharynx, as well as the upper, middle and lower pharyngeal airway width (UPAW, MPAW and LPAW). *Results*: Distinct sexual dimorphism was observed regarding the vertical cephalometric parameters within both Class I and Class III groups, with males exhibiting significantly larger facial height parameters, while females demonstrated larger nasopharyngeal depth angles (Ba–S–PNS). The Y–FH angle had significantly higher values in Class I than in Class III subjects, regardless of sex. Upper airway dimensions showed sexual dimorphism specifically in Class III subjects, with females exhibiting larger UPAW values than males. The inter-class comparisons showed larger values for LPAW, especially in females. Correlation analyses revealed no statistically significant relationships between the vertical and the upper airway parameters in Class I subjects. UPAW showed a tendency to decrease in Class III subjects as TAFH and LAFH increased. Ba–S–PNS showed consistent negative correlations with the vertical dimensions in both groups. *Conclusions:* These findings suggest that skeletal Class I and Class III malocclusions exhibit not only different sagittal relationships, but also distinctive, sex-related vertical skeletal patterns within each group, and therefore it would be advised that male and female patients should be diagnosed and treated according to separate protocols. In our population, Class III males are more likely to require orthognathic surgery, in addition to orthodontic treatment, with a more reserved prognosis and they might have a higher risk of OSA or other respiratory disorders in comparison with Class III females.

## 1. Introduction

Different types of malocclusions can develop from variations in craniofacial growth and development in the transverse, sagittal or vertical planes. The malocclusions in the sagittal plane are usually given priority in orthodontic treatment, because they can lead to substantial dento-facial alterations, significantly impacting the quality of life, with notable functional, aesthetic and psychological repercussions [1,2]. Sagittal skeletal discrepancies can be assessed using several cephalometric analyses, but Steiner’s ANB angle has proved to be a reliable and accurate diagnostic marker [3,4]. In order to better characterise the skeletal and dento-alveolar sagittal discrepancy, we also included the Wits appraisal in our analysis. According to the Wits appraisal and the ANB angle values, we can classify the sagittal skeletal malocclusions in three classes. Skeletal Class I malocclusion is defined as a relatively normal relationship in the sagittal plane between the upper and the lower jaws, but in which the teeth are malpositioned. Skeletal Class II malocclusion is characterised by a retrognathic mandible in relation to the maxilla, resulting in a convex facial profile. Conversely, skeletal Class III malocclusion is characterised by a prognathic mandible in relation to the maxilla, resulting in a concave facial profile.

In addition to normal sagittal relationships, balanced vertical skeletal proportions have an essential role in craniofacial aesthetics [5,6,7,8]. Moreover, varied interpretations of the cephalometric measurements used to assess the vertical craniofacial patterns may lead to different treatment strategies and outcomes [9].

The vertical skeletal pattern can be assessed using several measurements: the total anterior facial height, which has two components (the lower and upper anterior facial height), and the total posterior facial height [10].

Adequate tools for early diagnosis may be available to clinicians if they can recognise the potential interplay between the sagittal and vertical cephalometric features and the respiratory function. This would be particularly helpful in cases with a predisposition to conditions like obstructive sleep apnoea (OSA), or in cases with severe skeletal discrepancies that require orthognathic surgery, which in turn might affect the morphology of the upper airway by reducing its volume and potentially leading to OSA, although the results in the literature are conflicting. Therefore, the correlation between the upper airway morphology and the dimensional variations of the craniofacial structures during the stage of growth and development has been a subject of prolonged debate in the scientific literature. Some studies reported notable relationships [11,12,13,14,15,16,17,18], whereas others identified no connection between different craniofacial types and the dimensions of the segments of the upper airway [19,20,21,22,23,24,25,26].

Being part of the upper airways, the pharynx is a musculomembranous funnel-shaped hollow organ divided into three anatomical regions: the nasopharynx—the uppermost section, that connects to the nasal passages; the oropharynx—located in the middle section of the pharynx, delimited by the soft palate (cranially) and by the epiglottis (caudally); and the hypopharynx (laryngopharynx)—the lowermost segment, delimited by the epiglottis (cranially) and the oesophagus (caudally).

Digital lateral cephalograms are effective means of evaluating both the vertical and the sagittal soft tissue, dental and skeletal discrepancies. Even though these complementary exams do not offer data regarding the volume or the depth of anatomical structures, they provide valuable and reliable data about the craniofacial and upper airway morphologies, as well as their relationship to surrounding tissues, with minimal exposure to ionising radiation [27,28].

The literature proposes various cephalometric landmarks to define the aforementioned sections and segments. However, in order to be consistent, in the present study, we used a similar approach as in previously published studies, in which we compared groups of patients with skeletal Class I and skeletal Class II malocclusion [29,30].

### Aim and Objectives

The main objectives of our study were to assess sexual dimorphism and to compare the facial height, as well as the anteroposterior width of the upper airways, within adult Romanian Caucasians with skeletal Class I and skeletal Class III malocclusion, based on a number of angular and linear cephalometric parameters.

## 2. Materials and Methods

### 2.1. Study Protocol

A total of 100 lateral cephalograms were obtained from patient records in the Orthodontics II Department, Faculty of Dental Medicine, “Victor Babes” University of Medicine and Pharmacy of Timisoara, as well as from our private clinic. The adult patients included in this retrospective observational study had an average age of 24.7 years (age range—18 to 36 years). Based on the Wits appraisal and on Steiner’s ANB angle, the patients were diagnosed with skeletal Class I and Class III malocclusion. Each skeletal group included 25 females and 25 males. All the subjects consented in writing to take part in medical research. The Ethics Committee of “Victor Babes” University of Medicine and Pharmacy of Timisoara approved the present study, which was carried out in compliance with the ethical principles stipulated under the Declaration of Helsinki. The following inclusion criteria were used: Romanian Caucasian adults; subjects exhibiting Wits values of 0 ± 1 mm in females, −1 ± 1 mm in males and ANB = 2° ± 2° were included in the skeletal Class I group; subjects exhibiting Wits values of less than −1 mm in females, less than −2 mm in males and ANB < 0° were included in the skeletal Class III group. The exclusion criteria were as follows: poor-quality cephalograms, not Caucasian, medical history showing prior orthognathic surgery or prior orthodontic treatment, respiratory disease, the obstruction of the upper airway, extracted or missing posterior teeth (with the exception of the third molars). During the radiological examination, the subjects were advised not to swallow and to maintain a normal resting position of the tongue.

AudaxCeph 5 Essentials software (v. 5.2.0.3610) was used to trace and analyse the lateral teleradiographs, using a number of angular and linear cephalometric parameters. The analysis included several skeletal cephalometric landmarks: porion (Po), orbitale (Or), nasion (N), Point A, Point B, sella (S), articulare (Ar), basion (Ba), anterior nasal spine (ANS), the perpendicular projection of the skeletal point ANS onto the N–Me line (prANS), posterior nasal spine (PNS), gonion tangent point (tGo), menton (Me) and gnathion (Gn).

**Figure 1:**
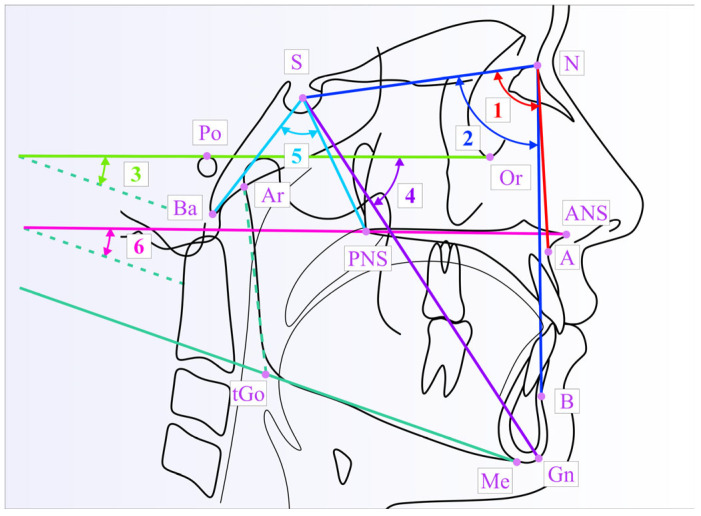
The angular measurements illustrated on the cephalometric tracing: SNA (1); SNB (2); FMA (3); Y–FH (4); Ba–S–PNS (5) and NL–ML (6).

The Frankfort–mandibular plane angle (FMA), formed by the intersection of the mandibular plane (Me–tGo) and the Frankfort horizontal plane (Or–Po), was used to assess the vertical facial types in conjunction with the measurements of facial height. In order to assess the growth direction of the mandible, we used the angle between the Y-axis (S–Gn) and the Frankfort horizontal plane (FH). The Ba–S–PNS angle was used to assess the nasopharyngeal depth. In order to assess the vertical skeletal relationship, especially in relation to the lower anterior facial height, we also used the NL–ML angle formed by the bispinal plane (ANS–PNS) and the mandibular plane (tGo–Me). Figure 1 illustrates the angular parameters on the cephalometric tracing.

**Figure 2:**
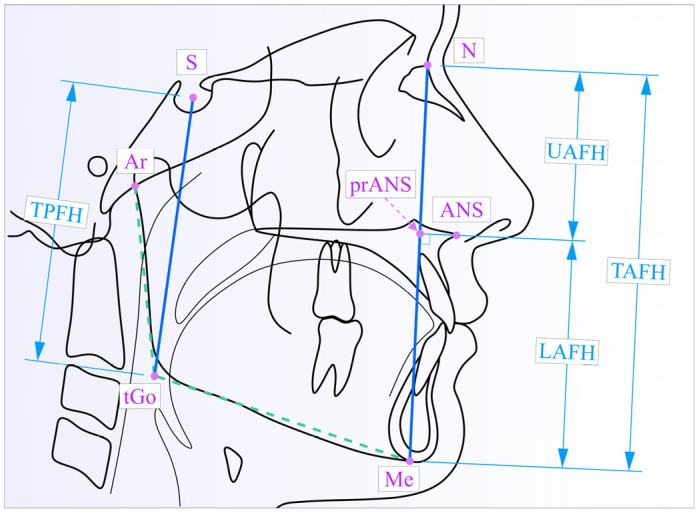
The vertical parameters illustrated on the cephalometric tracing: S–tGo (TPFH); N–Me (TAFH); N–prANS (UAFH) and prANS–Me (LAFH).

The template in Figure 2 illustrates all the cephalometric landmarks and the vertical linear measurements used to evaluate the total, upper and lower anterior facial height (TAFH, UAFH and LAFH, respectively) and the total posterior facial height (TPFH), as described in a previous study [29].

In order to evaluate the width of the upper airway, several cephalometric landmarks were used, following a methodology similar to the one presented in another published study of ours [30].

**Figure 3:**
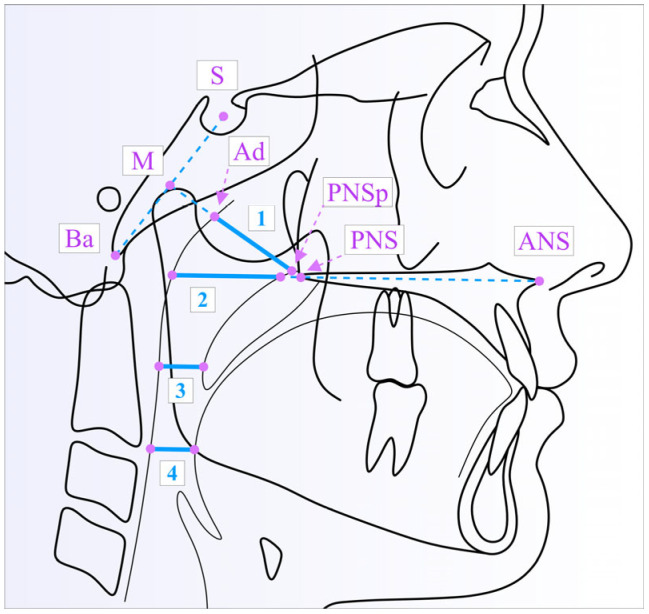
The upper airway and the respective linear measurements illustrated on the cephalometric tracing: PNSp–Ad (1); UPAW (2); MPAW (3); LPAW (4).

The cephalometric tracing with the linear measurements of the width of the nasopharynx (PNSp–Ad), as well as the upper, middle and lower pharyngeal airway width (UPAW, MPAW and LPAW, respectively), is illustrated in Figure 3. The constructed landmarks, PNSp and Ad, were derived from the intersection of the PNS–M line (M = midpoint of S–Ba) with the nasopharynx outline. The PNSp is located in the anteroinferior region, while the adenoid point (Ad) is situated in the posterosuperior area of the nasopharynx. UPAW, MPAW and LPAW were measured along parallel lines to the bispinal plane (ANS–PNS).

### 2.2. Statistical Analysis

We used the IBM SPSS Statistics software for Windows (Version 25.0, Armonk, NY, USA: IBM Corp.) to statistically analyse the data and to compile descriptive statistics for the cephalometric parameters. In order to test for differences between the examined groups, we used independent-sample *t*-tests for the data that were normally distributed (as demonstrated by the Shapiro–Wilk test). Non-parametric Mann–Whitney U tests were used to analyse the data that did not follow a normal distribution. Pearson correlation coefficients were calculated to test whether there were any linear relationships between the normally distributed variables, while Spearman’s rank correlation coefficients were used for the data that were not normally distributed. The level of significance was set at *p* < 0.05.

Thirty cephalograms were randomly selected and were reassessed after one month by the same examiner (GP). In order to determine the intrarater reliability, we calculated the intra-class correlation coefficient (ICC) using the IBM SPSS Statistics software package based on a two-way mixed-effects model with absolute agreement and a mean rating (k = 2).

## 3. Results

### 3.1. Reliability of Measurements

We found a high degree of reliability between the first and the second measurement. The average measured intra-class correlation coefficient (ICC) was 0.99, with a 95% confidence interval from 0.979 to 0.995 (*p* < 0.001).

### 3.2. Comparison Between Groups

The descriptive statistics for the measured variables and the *p*-values for the statistical tests (two-tailed independent *t*-test and Mann–Whitney U test) that were used to compare the differences between female and male subjects in the Class I and Class III groups are presented in Table 1.

The Y–FH angle in the Class I group showed higher mean values in all inter-class comparisons for females (*p* = 0.022), males (*p* = 0.032) and for the entire Class I group, when compared with the entire Class III group (*p* = 0.002).

When comparing the vertical cephalometric parameters between females and males within their respective groups, the males had significantly higher mean values than the females both in Class I (TAFH, *p* < 0.001; UAFH, *p* = 0.001; LAFH, *p* < 0.001; TPFH, *p* < 0.001; TPFH/TAFH, *p* < 0.001) and in Class III (TAFH, *p* < 0.001; UAFH, *p* < 0.001; LAFH, *p* < 0.001; TPFH, *p* < 0.001).

No statistically significant differences were found in the vertical cephalometric parameters between the inter-class comparisons, with the exception of UAFH (*p* = 0.030), which had higher mean values in Class III males than in Class I males.

When comparing the upper airway parameters between females and males within their respective groups, the females had significantly higher mean values than the males for the Ba–S–PNS angle in both Class I (*p* = 0.021) and Class III (*p* = 0.006). UPAW had significantly lower mean values in Class III males when compared to both Class III females (*p* = 0.003) and Class I males (*p* = 0.021).

The inter-class comparisons for LPAW showed significantly higher mean values for both Class III females when compared to Class I females (*p* = 0.032) and for the entire Class III group when compared to the entire Class I group (*p* = 0.016).

### 3.3. Correlation Coefficients

The guidelines for the interpretation of correlation coefficients are shown in Table 2.

The Pearson correlation coefficients (*r*) for the normally distributed datasets, the Spearman rank correlation coefficients (*rho*) for the variables that were not normally distributed and their corresponding *p*-values are presented in Table 3 for the skeletal Class I group and in Table 4 for the skeletal Class III group.

The following statistically significant correlations were found in the skeletal Class I group:

▪ Strong positive correlations:
  — FMA and NL–ML (*rho* = 0.814, *p* < 0.001);
  — TAFH and TPFH (*r* = 0.783, *p* < 0.001).
▪ Moderate positive correlations:
  — Y–FH with FMA (*rho* = 0.630, *p* < 0.001), NL–ML (*r* = 0.542, *p* < 0.001), TAFH (*r* = 0.453, *p* = 0.001) and LAFH (*r* = 0.501, *p* < 0.001);
  — NL–ML and LAFH (*r* = 0.523, *p* < 0.001);
  — TPFH with UAFH (*r* = 0.468, *p* = 0.001) and LAFH (*r* = 0.694, *p* < 0.001);
  — PNSp–Ad with UPAW (*rho* = 0.404, *p* = 0.004) and MPAW (*rho* = 0.447, *p* = 0.001);
  — LPAW and MPAW (*r* = 0.504, *p* < 0.001).
▪ Moderate negative correlations:
  — FMA and TPFH/TAFH (rho = −0.690, *p* < 0.001);
  — NL–ML with UAFH (*r* = −0.429, *p* = 0.002) and TPFH/TAFH (*r* = −0.615, *p* < 0.001);
  — Y–FH and Ba–S–PNS (*r* = −0.518, *p* < 0.001).
▪ Weak positive correlations:
  — FMA and LAFH (*rho* = 0.319, *p* = 0.024);
  — NL–ML with TAFH (*r* = 0.323, *p* = 0.022) and MPAW (*r* = −0.291, *p* = 0.040);
  — Ba–S–PNS and UPAW (*r* = 0.336, *p* = 0.017).
▪ Weak negative correlations:
  — FMA and TPFH (*rho* = −0.293, *p* = 0.039);
  — Ba–S–PNS with FMA (*rho* = −0.336, *p* = 0.017), TAFH (*r* = −0.381, *p* = 0.006), LAFH (*r* = −0.390, *p* = 0.005) and TPFH (*r* = −0.382, *p* = 0.006).

The following statistically significant correlations were found in the skeletal Class III group:

▪ Strong positive correlation:
  — FMA and NL–ML (*r* = 0.792, *p* < 0.001).
▪ Strong negative correlation:
  — TPFH/TAFH and NL–ML (*r* = −0.700, *p* < 0.001).
▪ Moderate positive correlations:
  — NL–ML and LAFH (*r* = 0.481, *p* < 0.001);
  — Y–FH with FMA (*r* = 0.601, *p* < 0.001), TAFH (*r* = 0.441, *p* = 0.001) and LAFH (*r* = 0.517, *p* < 0.001);
  — Ba–S–PNS and UPAW (*r* = 0.411, *p* = 0.003);
  — TPFH with TAFH (*r* = 0.651, *p* < 0.001), UAFH (*r* = 0.530, *p* < 0.001) and LAFH (*r* = 0.541, *p* < 0.001);
  — PNSp–Ad with UPAW (*r* = 0.590, *p* < 0.001) and MPAW (*r* = 0.422, *p* = 0.002);
  — LPAW and MPAW (*r* = 0.604, *p* < 0.001).
▪ Moderate negative correlations:
  — FMA and TPFH/TAFH (*r* = −0.697, *p* < 0.001);
  — Ba–S–PNS with TAFH (*r* = −0.436, *p* = 0.002) and TPFH (*r* = −0.528, *p* < 0.001).
▪ Weak positive correlations:
  — FMA with TAFH (*r* = 0.316, *p* = 0.025) and LAFH (*r* = 0.388, *p* = 0.005);
  — NL–ML with Y–FH (*r* = 0.361, *p* = 0.010) and TAFH (*r* = 0.299, *p* = 0.035);
  — UAFH and LAFH (*r* = 0.332, *p* = 0.018).
▪ Weak negative correlations:
  — FMA and TPFH (*r* = −0.299, *p* = 0.035);
  — NL–ML and TPFH (*r* = −0.315, *p* = 0.026);
  — Ba–S–PNS with Y–FH (*r* = −0.332, *p* = 0.018), UAFH (*r* = −0.330, *p* = 0.019) and LAFH (*r* = −0.379, *p* = 0.007);
  — UPAW with TAFH (*r* = −0.359, *p* = 0.010) and LAFH (*r* = −0.387, *p* = 0.006).

## 4. Discussion

Different authors describe multiple methods and landmarks for evaluating the differences and/or the relationships between the cephalometric skeletal components and the characteristics of the upper airways, which leads to a real challenge in comparing the findings reported in the literature, especially when different racial and ethnic backgrounds are considered. Many studies related to various cephalometric parameters in skeletal Class III malocclusion are conducted on young patients, in contrast with our study, which only included adult subjects. Moreover, very few studies include correlation analyses between these parameters, therefore limiting the comparability of our results with other studies.

It has been long debated whether the morphology of the upper airway is correlated with or influenced by the anatomical characteristics of the craniofacial structures. In the present study, we focused on these potential correlations, as some sagittal and/or vertical skeletal patterns might be more predisposed to respiratory disorders, including OSA. In this regard, severe cases of Class III malocclusion, which require mandibular setback, might be particularly affected, as the upper airway space can be reduced by the posterior displacement of the mandible.

Yavari et al. reported that a small mandibular setback (under 5 mm) will not increase the risk of obstructive sleep apnoea in young adults with Class III malocclusion, but that the risk increases in larger setbacks [32]. Similar results were found by Eriksen et al., who stated that a mandibular setback under 7.4 mm did not lead to OSA [33].

Following a systematic review, Indriksone and Jakobsone [26] concluded that nearly 50% of the studies found no significant differences in the anterior–posterior linear measurements and in the volume of the oropharyngeal airway, while 75% of the studies found no significant differences in the dimensions of the nasopharyngeal airway when comparing different skeletal sagittal patterns. Six of eleven studies reported that the oropharyngeal dimensions in Class I and Class II groups were smaller than in Class III. The authors also concluded that the evidence was insufficient to substantiate the hypothesis that different sagittal skeletal patterns correlate with different dimensions of the upper airways.

In a following study, the same authors reported that the craniofacial structures did not significantly influence the morphology of the upper airway, when analysing a sample group of 276 patients, aged 17 to 27 years [34].

Most studies on Caucasian populations failed to demonstrate any significant correlations between the dimension or volume of the upper airway and the craniofacial skeletal patterns [25,34]. On the other hand, Zheng et al. [18], in a CBCT study conducted on an Asian population with an average age of 15.6 years, found that the narrowest regions and the volume of the upper respiratory tract varied across different sagittal skeletal types; Class I and Class III patients had larger nasopharyngeal airways compared to those with Class II skeletal malocclusion. Other authors reported that skeletal Class III subjects generally had wider UPAW and LPAW compared to skeletal Class I and Class II patients [35,36].

Our study was partially in agreement with these findings, with regard to LPAW, especially in female subjects, whereas Class III male subjects exhibited narrower UPAW when compared to the males in the skeletal Class I group.

According to Altheer et al. [37], in a systematic review that included 66 articles published in several main scientific databases, 19 articles were found that studied the volume of the oropharynx in 2035 patients, showing significant differences between the three classes of malocclusion. It was reported that the dimensions of the oropharynx in Class III patients were larger than in both Class I and Class II cases.

Our study found no statistically significant correlations between the FMA angle and the pharyngeal airway dimensions in Class III subjects, but we found negative correlations between UPAW and two vertical parameters, TAFH and LAFH. These findings suggest that UPAW tends to decrease in Class III subjects as TAFH and LAFH increase. Several studies showed that hyperdivergent (high-angle) growth patterns are associated with narrower upper pharyngeal airways across all skeletal classes [38,39,40].

These results suggest that the Class III males in our population, which showed narrower upper pharyngeal airways, are at higher risk of OSA or other respiratory disorders in comparison with Class III females, especially when mandibular setback orthognathic surgery is required.

In the present study, both in skeletal Class I and Class III groups, the FMA angle and the maxillomandibular plane angle (NL–ML) were inversely correlated with the Jarabak index (TPFH/TAFH), meaning that as one cephalometric parameter increases, the other has a tendency to decrease. This relationship was consistent across different studies, making it a reliable finding for assessing the skeletal growth pattern [41,42]. Both skeletal Class I and Class III groups also showed strong positive correlations between the FMA and NL–ML angles.

All the investigated groups (skeletal Class I, II and III malocclusion) were reported by Jain et al. to exhibit sexual dimorphism, which was demonstrated by several pharyngeal characteristics [43]. Males were reported to have wider lower pharyngeal airways than females in the skeletal Class III group. When compared to the skeletal Class I and II groups, LPAW was shown to be significantly larger in both males and females in the skeletal Class III group [43].

Analysing facial height, Obaidi [44] found no significant differences that could indicate sexual dimorphism, but found variations in facial height between the three dentoskeletal groups. TPFH had significantly lower values in the Class III group than in the Class I group [44].

Several authors [45,46,47] reported an increased LAFH in Class III patients, whereas some authors found reduced values for LAFH in Class III individuals [48,49]. Ellis and McNamara [45], in a study on 302 adult patients of 17 years or older, also reported significant sexual dimorphism regarding LAFH, which was higher in men, similar to our findings. Very few studies reported a decreased LAFH in Class III malocclusion, especially in Caucasian adults. De Frutos-Valle et al. analysed 212 Class III adult subjects originating from southern Europe and found one particular subphenotypic variation (representing 3.77% from the sample group) which was characterised by decreased TPFH, and another subphenotype (16%) characterised by decreased TAFH and LAFH [48].

In the present study, we observed no statistically significant differences in the vertical parameters between the two skeletal groups when compared overall, with the exception of UAFH, which was greater in Class III males than in Class I males. Sexual dimorphism was evident across both the Class I and Class III groups, the male subjects showing larger facial height measurements. Our findings aligned with those published by Baccetti, Reyes and McNamara [50], who concluded that craniofacial parameters in Class III malocclusion exhibited a significant degree of sexual dimorphism, the males showing significantly larger anterior facial height than the females, especially during or after puberty, extending into adulthood. The study was conducted on 1094 pretreatment lateral cephalograms obtained from Caucasian subjects diagnosed with Class III malocclusion, 3 to 57 years old, divided into twelve age groups.

Contrary to our findings, a recent study, conducted on 75 Class III Romanian Caucasians, found no sexual dimorphism regarding TAFH, TPFH and the Jarabak ratio [51]. The Class III group included patients aged 8 to 20 years and was divided into two separate age groups, in order to characterise the changes before and after the pubertal growth stage [51].

From a clinical and practical point of view, most authors agree that a vertical growth pattern in Class III malocclusion is correlated with an unfavourable prognosis and treatment outcome [52,53], most cases requiring a combined orthodontic and orthognathic surgery approach [45]. Therefore, we can conclude that, at least in our population, Class III males are more likely to require orthognathic surgery, in addition to orthodontic treatment, with a more reserved prognosis in comparison with Class III females.

A limitation of this study is that the classification according to Steiner’s ANB angle and the Wits appraisal did not distinguish between Class III skeletal anomalies caused by mandibular prognathism, maxillary retrognathism or a combination of both.

Acknowledging the limitations of 2D radiographic investigations, in order to better understand the anatomical features of the craniofacial structures, we plan on broadening our future research to also include CBCT scans for assessing the volume of the upper respiratory tract and to increase the sample size to improve the precision of the estimates in the studied population.

## 5. Conclusions

Distinct sexual dimorphism was observed regarding the vertical cephalometric parameters within both Class I and Class III groups, with males showing significantly larger measurements in total, upper and lower anterior facial height, as well as in total posterior facial height. However, no statistically significant differences were found in the vertical cephalometric parameters between the two skeletal groups, with the exception of the upper anterior facial height, which was greater in Class III males than in Class I males. In our population, Class III males are more likely to require orthognathic surgery, in addition to orthodontic treatment, with a more reserved prognosis in comparison with Class III females.

The Y–FH angle, as an indicator of the direction of facial growth, showed significantly higher values in Class I than in Class III subjects, regardless of sex, suggesting potentially different growth patterns in these types of malocclusions.

Upper airway dimensions showed sexual dimorphism specifically in Class III subjects, with females exhibiting a larger upper pharyngeal airway width than males, while this difference was not observed in Class I subjects. The inter-class comparisons showed that the Class III group had a larger lower pharyngeal airway width, especially in females. The females also demonstrated consistently larger nasopharyngeal depth angles in both skeletal groups. This suggests that Class III males are at higher risk of OSA or other respiratory disorders in comparison with Class III females, especially when mandibular setback orthognathic surgery is required.

Correlation analyses revealed no statistically significant relationships between the vertical and the upper airway parameters in Class I subjects. The width of the upper pharyngeal airways showed a tendency to decrease in Class III subjects as the total and lower anterior facial height increased. The nasopharyngeal depth angle showed consistent negative correlations with the vertical dimensions in both groups.

These findings suggest that skeletal Class I and Class III malocclusions exhibit not only different sagittal relationships, but also distinctive, sex-related vertical skeletal patterns within each group, and to a lesser extent, sex-related differences in upper airway width; therefore, it would be advised that male and female patients should be diagnosed and treated according to separate protocols.

## Figures and Tables

**Table 1:** Descriptive statistics for the measured cephalometric parameters and the *p*-values for the statistical tests used to compare the differences between female and male subjects in Class I and Class III.

				Descriptive Statistics		Independent *t*-Test/^b^ Mann–Whitney U Test Sig. (2-Tailed)				
Variables	Unit of Measure			Class I	Class III	Class I	Class III	Class I vs. Class III		
		Sex	N	Mean ± SD ^a^ Median ± IR		F vs. M	F vs. M	F	M	T
Age	years	F	25	23.68 ± 3.11	25.04 ± 4.67	-	-	-	-	-
		M	25	25.76 ± 4.25	24.44 ± 3.45					
		T	50	24.72 ± 3.83	24.74 ± 4.08					
ANB	deg	F	25	2.33 ± 0.96	−2.38 ± 1.92	-	-	-	-	-
		M	25	2.11 ± 1.03	−2.34 ± 1.80					
		T	50	2.22 ± 0.99	−2.36 ± 1.84					
Wits	mm	F	25	−0.14 ± 1.02	−4.85 ± 2.90	-	-	-	-	-
		M	25	−0.32 ± 0.76	−5.06 ± 2.99					
		T	50	−0.23 ± 0.89	−4.96 ± 2.92					
FMA	deg	F	25	23.06 ± 3.93	21.62 ± 5.18	0.184 ^b^	0.548	0.273	0.720 ^b^	0.298 ^b^
		M	25	21.37 ± 5.69 ^a^	20.88 ± 3.26					
		T	50	21.88 ± 5.86 ^a^	21.25 ± 4.30					
NL–ML	deg	F	25	22.78 ± 5.64	21.63 ± 4.92	0.642	0.622	0.445	0.295	0.214
		M	25	22.15 ± 3.79	21.01 ± 3.78					
		T	50	22.46 ± 4.77	21.32 ± 4.35					
Y–FH	deg	F	25	58.55 ± 2.49	56.54 ± 3.43	0.492	0.371	0.022 *	0.032 *	0.002 **
		M	25	59.14 ± 3.42	57.30 ± 2.37					
		T	50	58.84 ± 2.98	56.92 ± 2.94					
Ba–S–PNS	deg	F	25	58.27 ± 3.14	57.15 ± 3.51	0.021 *	0.006 **	0.242	0.235	0.119
		M	25	55.47 ± 4.98	53.85 ± 4.50					
		T	50	56.87 ± 4.36	55.50 ± 4.33					
TAFH	mm	F	25	108.92 ± 4.08	109.44 ± 6.09	0.000 ***	0.000 ***	0.727	0.744	0.973
		M	25	118.70 ± 5.16	118.28 ± 3.86					
		T	50	113.81 ± 6.75	113.86 ± 6.74					
UAFH	mm	F	25	48.82 ± 2.46	48.53 ± 2.78	0.001 ***	0.000 ***	0.705	0.030 *	0.324
		M	25	50.90 ± 1.60	52.32 ± 2.71					
		T	50	49.86 ± 2.30	50.43 ± 3.32					
LAFH	mm	F	25	60.11 ± 4.46	60.90 ± 4.76	0.000 ***	0.000 ***	0.545	0.135	0.636
		M	25	67.80 ± 4.94	65.96 ± 3.50					
		T	50	63.95 ± 6.07	63.43 ± 4.86					
TPFH	mm	F	25	72.94 ± 3.53	74.95 ± 4.75	0.000 ***	0.000 ***	0.096	0.672	0.591
		M	25	84.31 ± 4.20	83.75 ± 4.98					
		T	50	78.62 ± 6.91	79.35 ± 6.56					
TPFH/TAFH	%	F	25	67.01 ± 3.20	68.59 ± 4.32	0.000 ***	0.067	0.148	0.825	0.414
		M	25	71.08 ± 3.31	70.84 ± 4.17					
		T	50	69.04 ± 3.82	69.72 ± 4.35					
PNSp–Ad	mm	F	25	17.98 ± 3.05	18.85 ± 3.53	0.648 ^b^	0.584	0.352	0.866	0.567 ^b^
		M	25	18.60 ± 3.38 ^a^	18.31 ± 3.44					
		T	50	18.55 ± 4.14 ^a^	18.58 ± 3.46					
UPAW	mm	F	25	20.40 ± 1.91	20.64 ± 2.98	0.326	0.003 **	0.736	0.021 *	0.190
		M	25	19.82 ± 2.22	18.22 ± 2.51					
		T	50	20.11 ± 2.07	19.43 ± 2.99					
MPAW	mm	F	25	8.85 ± 2.19	9.86 ± 2.73	0.681	0.098	0.156	0.490	0.566
		M	25	9.12 ± 2.35	8.66 ± 2.27					
		T	50	8.99 ± 2.25	9.26 ± 2.56					
LPAW	mm	F	25	10.18 ± 2.68	12.03 ± 3.22	0.074	0.576	0.032 *	0.209	0.016 *
		M	25	11.48 ± 2.34	12.57 ± 3.60					
		T	50	10.83 ± 2.57	12.30 ± 3.39					

F—female; M—male; T—total; SD—standard deviation; IR—interquartile range; Sig.—significance level; **^a^** Median ± IR; ^b^ Mann–Whitney U Test Sig. (2-Tailed); * *p* < 0.05; ** *p* < 0.01; *** *p* < 0.001.

**Table 2:** Guidelines for the interpretation of correlation coefficients. Adapted after Schober, Boer and Schwarte [31].

Coefficient Value	Relationship Strength/Correlation
0.00 to ±0.09	Negligible
±0.10 to ±0.39	Weak
±0.40 to ±0.69	Moderate
±0.70 to ±0.89	Strong
±0.90 to ±0.99	Very strong
1	Perfect

Colour codes for the strength of correlation: blue—weak, green—moderate, gold—strong and orange—very strong.

**Table 3:** Pearson correlation coefficients (*r*)/Spearman rank correlation coefficients (*rho*) and the corresponding *p*-values calculated for the measured cephalometric parameters in the skeletal Class I group.

Class I Variables		FMA	NL–ML	Y–FH	Ba–S–PNS	TAFH	UAFH	LAFH	TPFH	TPFH/TAFH	PNSp–Ad	UPAW	MPAW	LPAW
FMA	CC	1												
	*p*													
NL–ML	CC	0.814 ^a^	1											
	*p*	0.000 **												
Y–FH	CC	0.630 ^a^	0.542	1										
	*p*	0.000 **	0.000 **											
Ba–S–PNS	CC	−0.336 ^a^	−0.270	−0.518	1									
	*p*	0.017 *	0.058	0.000 **										
TAFH	CC	0.240 ^a^	0.323	0.453	−0.381	1								
	*p*	0.093	0.022 *	0.001 **	0.006 **									
UAFH	CC	−0.190 ^a^	−0.429	0.009	−0.089	-	1							
	*p*	0.185	0.002**	0.952	0.537	-								
LAFH	CC	0.319 ^a^	0.523	0.501	−0.390	-	0.124	1						
	*p*	0.024 *	0.000 **	0.000 **	0.005 **	-	0.390							
TPFH	CC	−0.293 ^a^	−0.168	0.216	−0.382	0.783	0.468	0.694	1					
	*p*	0.039 *	0.245	0.133	0.006 **	0.000 **	0.001 **	0.000 **						
TPFH/TAFH	CC	−0.690 ^a^	−0.615	−0.144	−0.198	-	-	-	-	1				
	*p*	0.000 **	0.000 **	0.319	0.167	-	-	-	-					
PNSp–Ad	CC	−0.120 ^a^	−0.138 ^a^	−0.030 ^a^	0.080 ^a^	−0.103 ^a^	0.035 ^a^	−0.124 ^a^	−0.012 ^a^	0.157 ^a^	1			
	*p*	0.408	0.338	0.834	0.581	0.475	0.812	0.391	0.936	0.277				
UPAW	CC	−0.213 ^a^	−0.245	−0.177	0.336	−0.221	0.027	−0.256	−0.058	0.135	0.404 ^a^	1		
	*p*	0.138	0.086	0.218	0.017 *	0.124	0.851	0.073	0.689	0.350	0.004 **			
MPAW	CC	−0.271 ^a^	−0.291	−0.207	0.262	0.019	0.184	−0.048	0.142	0.200	0.447 ^a^	−0.012	1	
	*p*	0.057	0.040 *	0.149	0.066	0.894	0.202	0.740	0.324	0.163	0.001 **	0.937		
LPAW	CC	−0.069 ^a^	0.022	−0.123	−0.011	0.219	0.069	0.217	0.233	0.139	−0.002 ^a^	−0.148	0.504	1
	*p*	0.633	0.877	0.395	0.939	0.126	0.633	0.129	0.103	0.334	0.988	0.304	0.000 **	

CC—correlation coefficient; ^a^ Spearman rank correlation coefficient (*rho*). Refer to Table 2 for the colour codes used for the strength of correlation when * *p* < 0.05 or ** *p* < 0.01.

**Table 4:** Pearson correlation coefficients (*r*) and the corresponding *p*-values calculated for the measured cephalometric parameters in the skeletal Class III group.

Class III Variables		FMA	NL–ML	Y–FH	Ba–S–PNS	TAFH	UAFH	LAFH	TPFH	TPFH/TAFH	PNSp–Ad	UPAW	MPAW	LPAW
FMA	CC	1												
	*p*													
NL–ML	CC	0.792	1											
	*p*	0.000 **												
Y–FH	CC	0.601	0.361	1										
	*p*	0.000 **	0.010 **											
Ba–S–PNS	CC	−0.050	−0.042	−0.332	1									
	*p*	0.732	0.770	0.018 *										
TAFH	CC	0.316	0.299	0.441	−0.436	1								
	*p*	0.025 *	0.035 *	0.001 **	0.002 **									
UAFH	CC	0.074	−0.098	0.138	−0.330	-	1							
	*p*	0.611	0.499	0.340	0.019 *	-								
LAFH	CC	0.388	0.481	0.517	−0.379	-	0.332	1						
	*p*	0.005 **	0.000 **	0.000 **	0.007 **	-	0.018 *							
TPFH	CC	−0.299	−0.315	0.246	−0.528	0.651	0.530	0.541	1					
	*p*	0.035 *	0.026 *	0.085	0.000 **	0.000 **	0.000 **	0.000 **						
TPFH/TAFH	CC	−0.697	−0.700	−0.106	−0.267	-	-	-	-	1				
	*p*	0.000 **	0.000 **	0.464	0.061	-	-	-	-					
PNSp–Ad	CC	0.040	−0.037	0.012	0.225	−0.101	0.069	−0.187	−0.045	0.051	1			
	*p*	0.781	0.798	0.933	0.116	0.485	0.633	0.193	0.759	0.724				
UPAW	CC	0.033	−0.143	0.030	0.411	−0.359	−0.162	−0.387	−0.218	0.062	0.590	1		
	*p*	0.821	0.322	0.834	0.003 **	0.010 *	0.260	0.006 **	0.129	0.671	0.000 **			
MPAW	CC	−0.162	−0.194	−0.135	0.170	−0.228	−0.195	−0.183	0.000	0.228	0.422	0.161	1	
	*p*	0.262	0.177	0.349	0.238	0.110	0.174	0.203	1.000	0.112	0.002 **	0.265		
LPAW	CC	−0.160	−0.090	−0.049	0.057	0.055	0.018	0.064	0.182	0.197	0.202	−0.052	0.604	1
	*p*	0.268	0.533	0.734	0.694	0.705	0.900	0.660	0.206	0.171	0.160	0.721	0.000 **	

CC—correlation coefficient. Refer to Table 2 for the colour codes used for the strength of correlation when * *p* < 0.05 or ** *p* < 0.01.

## Data Availability

Additional data supporting the reported results can be requested from the corresponding authors.
